# Uniform Diffracted Fields from a Perfectly Conducting Cylindrical Reflector with Modified Theory of Physical Optics

**DOI:** 10.1155/2013/195402

**Published:** 2013-05-26

**Authors:** Uğur Yalçın, Mücahit Sarnık

**Affiliations:** Electronic Engineering Department, Faculty of Engineering and Architecture, Uludağ University, Görükle, 16059 Bursa, Turkey

## Abstract

The uniform diffracted fields are calculated on PEC cylindrical reflector by Modified Theory of Physical Optics (MTPO). It is aimed to convert the noncontinuous solution to a continuous solution by finding a uniform equation which does not contain any expression converging to 0 in the denominator part. Three axioms of MTPO theory are used to construct the integral equations for the perfectly electrically conducting surface application. The “edge-point” technique is used to find the diffracted field, and uniform solution is to be found via “detour parameter(s).” Finally, the obtained results are to be compared with the nonuniform ones, numerically.

## 1. Introduction

Physical Optics (PO) is a high frequency method to calculate the reflected and diffracted fields via induced current on a scatterer surface [1, 2]. The incident magnetic field is used to obtain the induced current [3]. However, there exists deficiency while obtaining edge-diffracted field [4]. The PO application of the black half plane surface is a typical example of such a deficiency [5].

The two following points can be defined as the cause of this lack. Firstly, the induced current is assumed on the reflection surface, whereas the aperture surface is not taken into account. Thus, the incident diffracted fields cannot be obtained due to this neglecting. Secondly, the edges are considered as continuous which cause to suppose the equality of reflection and incidence angle in the edge point.

Consequently, the innovative method of Modified Theory of Physical Optics (MTPO) has been derived in order to find the exact solution analytically on edge diffraction and to overcome the deficiencies of PO for edge currents [6]. The result is coherent with the solution of the exact solution of the Helmholtz equation [3]. MTPO was derived by Umul in 2004 via the paper about the scattering of the field from a “perfectly conducting” (PEC) half-plane surface [7]. Afterwards, the studies for the black half plane [8] and for the impedance half plane [9] were carried out by Umul.

Moreover, the study for “perfectly conducting cylindrical reflector” was fulfilled by Yalçın in 2007 [10], and the study for the scattering from “cylindrical parabolic impedance surface” was studied by Umul in 2008 [11]. Lastly, a brief explanation of MTPO and the synthesis of the studies have been presented [12].

There are three axioms of MTPO which are used to evaluate the asymptotic evaluation of scattering integral. First axiom is related to the induced current on a scatterer surface by which the reflected and reflected diffracted fields are obtained. However the incident diffracted field cannot be found. Hence, a second surface is assumed which is called an aperture surface. The induced currents on this surface are defined by the aid of Equivalent Source Theory, and so the incident and incident diffracted fields are calculated. Second axiom is the reflection/transmission angle which depends on the real and aperture surface coordinates and which is a function of integral variables. Last axiom is the definition of the vectors—different from PO—which divide the angle between the incident and reflected fields and which are the factors used to evaluate the induced current [13]. Additionally in MTPO integral, the phase function is expressed as a new form [14].

In this study, the uniform diffracted fields are calculated on PEC cylindrical reflector. Previously, on this surface, diffracted fields have been obtained as a nonuniform solution where the field equation diverges to infinity and which lead to discontinuity in the analysis [10]. Thus, in this work it is aimed to construct a continuous solution by finding a uniform equation and also to compare numerically the diffracted fields on PEC cylindrical reflector for the noncontinuous and continuous solutions of MTPO method.

A time factor *e* ^*jωt*^ is assumed and suppressed throughout the paper.

## 2. MTPO Theory

**Figure 1:**
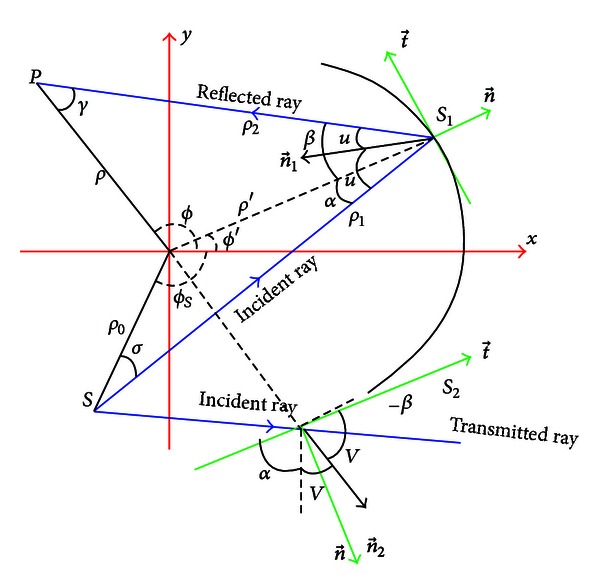
The *xy*-plane cross-section of the source and PEC cylinder.

For MTPO, induced currents on the real surface are defined as

(1a) $$\begin{array}{c} {\overset{-}{J}}_{\text{es}}={\left({\vec{n}}_{1}\times {\vec{H}}_{t}\right)|}_{S_{1}}, \end{array}$$

where ${\vec{H}}_{t}$ is the total magnetic field on the real surface (*S*_1_). The induced currents on the aperture surface (*S*_2_) are also calculated via the following formulas:

(1b) $$\begin{array}{c} {\overset{-}{J}}_{\text{es}}={\left({\vec{n}}_{2}\times {\vec{H}}_{i}\right)|}_{S_{2}}, \end{array}$$

(1c) $$\begin{array}{c} {\overset{-}{J}}_{\text{ms}}=-{\left({\vec{n}}_{2}\times {\vec{E}}_{i}\right)|}_{S_{2}}, \end{array}$$

where ${\vec{H}}_{i}$ denotes the incident magnetic field and ${\vec{E}}_{i}$ denotes the incident electrical field [7]. The vectors ${\vec{n}}_{1}$ and ${\vec{n}}_{2}$ are the ones mentioned in the third axiom of MTPO, and they are defined on the real (*S*_1_) and aperture (*S*_2_) surface, respectively. They can be formulized as

(2a) $$\begin{array}{c} {\vec{n}}_{1}=\sin\left(u-\alpha \right)\vec{t}-\cos\left(u-\alpha \right)\vec{n}, \end{array}$$

(2b) $$\begin{array}{c} {\vec{n}}_{2}=-\cos\left(v+\alpha \right)\vec{t}+\sin\left(v+\alpha \right)\vec{n}, \end{array}$$

where *α* denotes the incidence angle and $\vec{t}$ and $\vec{n}$ are the tangential and normal unit vectors of the surfaces, respectively, as they are shown in Figure 1. Moreover, *u* is equal to (*α* + *β*)/2, and *v* is equal to *π*/2 − (*α* − *β*)/2.

The total electrical field can be written as

(3) $$\begin{array}{c} {\vec{E}}_{t}={\vec{E}}_{\text{is}}+{\vec{E}}_{\text{rs}}, \end{array}$$

where incident scattered electrical field is

(4a) E→is=−jωμ04π∬Swn→2×H→i|S2e−jkR2R2 dS′+∬S2∇×(n→2×E→i|S2e−jkR2R2)dS′,

and reflecting scattered electrical field is

(4b) $$\begin{array}{c} {\vec{E}}_{\text{rs}}=-\frac{j\omega \mu_{0}}{4\pi }\iint_{S_{1}}^{}{{\vec{n}}_{1}\times {\vec{H}}_{t}|}_{S_{1}}\frac{e^{-jkR_{1}}}{R_{1}}\;dS^{\prime }. \end{array}$$

Then the related magnetic fields can also be formulized as

(5a) H→is=14π∬Sw∇×(n→2×H→i|S2e−jkR2R2)dS′+jωε4π∬S2n→2×E→i|S2e−jkR2R2 dS′,

(5b) $$\begin{array}{c} {\vec{H}}_{\text{rs}}=\frac{1}{4\pi }\iint_{S_{1}}^{}\nabla \times \left({{\vec{n}}_{1}\times {\vec{H}}_{t}|}_{S_{1}}\frac{e^{-jkR_{1}}}{R_{1}}\right)dS^{\prime }. \end{array}$$

## 3. Application

The incident electrical and magnetic fields from a line source are indicated as

(6a) $$\begin{array}{c} {\vec{E}}_{i}=-\frac{\omega \mu_{0}I}{4}H_{0}^{\left(2\right)}\left(k\rho_{1}\right){\vec{e}}_{z}, \end{array}$$

(6b) $$\begin{array}{c} {\vec{H}}_{i}=H_{i}\left(\sin\alpha {\vec{e}}_{\phi }+\cos\alpha {\vec{e}}_{\rho }\right), \end{array}$$

respectively. *H*_0_^(2)^(*kρ*_1_) is the Hankel function of the second kind where *k* is the number of wavelength and *ρ*_1_ is the distance between the source and the reflector. Therefore the reflected magnetic field is ${\vec{H}}_{r}=H_{i}(-\sin\beta {\vec{e}}_{\rho }+\cos\beta {\vec{e}}_{\phi })$. The total magnetic field which is used in the integral equation is obtained by

(7) $$\begin{array}{c} {\vec{H}}_{t}={\vec{H}}_{r}+{\vec{H}}_{i}=H_{i}\left(\sin\alpha {\vec{e}}_{\phi }+\cos\alpha {\vec{e}}_{\rho }-\sin\beta {\vec{e}}_{\rho }+\cos\beta {\vec{e}}_{\phi }\right). \end{array}$$

The magnitude of the incident ray is equal to *ρ*_1_ = [*ρ* ^′2^+*ρ*_0_^2^−2*ρ*′*ρ*_0_cos⁡(*ϕ*′−*ϕ*_*s*_)]^1/2^ according to Figure 1. The surface current induced on *S*_1_ is found as

(8) $$\begin{array}{c} {\vec{J}}_{\text{es}}=-{\vec{e}}_{z}2{\vec{H}}_{i}\cos\left(\frac{\alpha +\beta }{2}\right). \end{array}$$

The other surface currents which are induced on aperture surface are also calculated as

(9a) $$\begin{array}{c} {\vec{J}}_{\text{es}}={\vec{e}}_{z}{\vec{H}}_{i}\cos v, \end{array}$$

(9b) $$\begin{array}{c} {\vec{J}}_{\text{ms}}=-E_{i}\left[{\vec{e}}_{\rho }\sin\left(v+\alpha \right)-{\vec{e}}_{\phi }\cos\left(v+\alpha \right)\right]. \end{array}$$

The surface element can be shown as *dS*′ = *a* *dϕ*′ *dz*′, where the radius of the cylinder is *ρ*′ = *a*. By the Green function, which is equal to $G\left(\vec{r},{\vec{r}}^{\prime }\right)=e^{-jkR_{}}/R$, the equation can be written to solve the integral

(10) ∫z=−∞∞e−jkR1R1 dz′ =∫z′=−∞∞12j∫ζ=−∞∞H0(2)(k2−ζ2ρ1)e−jζ(z−z′) dζ  dz′.

By the property ∫_*z*′=−*∞*_^*∞*^ *e* ^*jζz*′^ *dz*′ = 2*πδ*(*ζ*), the Green function can be simplified to

(11) $$\begin{array}{c} \int_{z=-\infty }^{\infty }\frac{e^{-jkR_{1}}}{R_{1}}\;dz^{\prime }=\frac{\pi }{j}H_{0}^{\left(2\right)}\left(k\rho_{1}\right). \end{array}$$

By the utilization of (4a) and (4b) the total scattered field can be obtained as

(12) E→s=E→is+E→rs=−e→zk2Z0Ia8 ×{∫ϕ′=−ϕ0ϕ0cos⁡⁡(α+β2)H0(2)(kρ1)H0(2)(kρ2)dϕ′  +∫ϕ′=ϕ0−ϕ0sin(α−β2)H0(2)(kρ1)H0(2)(kρ2)dϕ′}.

By the Debye's asymptotic expansion of the Hankel function

(13) $$\begin{array}{c} H_{0}^{\left(2\right)}\left(k\upsilon \right)\approx \sqrt{\frac{2}{\pi }}\frac{e^{-jk\upsilon +j(\pi /4)}}{\sqrt{k\upsilon }}, \end{array}$$

the total scattered field equation is converted to

(14) E→s=−e→zkZ0Ia4π ×[∫ϕ′=−ϕ0ϕ0cos⁡⁡(α+β2)e−jk(ρ1+ρ2)ρ1ρ2 dϕ′+∫ϕ′=ϕ0−ϕ0sin⁡(α−β2)e−jk(ρ1+ρ2)ρ1ρ2 dϕ′].

Thus, the scattering integral equations, which are obtained by MTPO, are in a form of

(15) $$\begin{array}{c} I_{S}=\int_{x^{\prime }=-\infty }^{\infty }f\left(x^{\prime }\right)e^{-jk\psi (x^{\prime })}\;dx^{\prime }, \end{array}$$

where *f*(*x*′) is represented by the sinusoidal term and *ψ*(*x*′) is represented by (*ρ*_1_ + *ρ*_2_). The phase function has been found as

(16) $$\begin{array}{c} \psi \left(\phi^{\prime }\right)=\rho_{1}+\rho_{2}=\rho_{0}\cos\sigma +\rho^{\prime }\cos\alpha +\rho \cos\gamma +\rho^{\prime }\cos\beta \end{array}$$

by utilizing the angles and the distances shown Figure 1. If the phase function is derived with respect to *ϕ*′, the equation

(17) ψ′(ϕ′)=−ρ0sinσdσdϕ′−ρ′sinαdαdϕ′−ρsinγdγdϕ′−ρ′sinβdβdϕ′

can be obtained. The angles can be rewritten by Figure 1 as follows:

(18a) $$\begin{array}{c} \sigma =\pi \pm \phi_{s}\mp \phi^{\prime }-\alpha , \end{array}$$

(18b) $$\begin{array}{c} \gamma =\pi \mp \phi_{s}\pm \phi^{\prime }-\beta . \end{array}$$

By the sine relations, the following equations are obtained:

(19) $$\begin{array}{c} \frac{\rho_{0}}{\sin\alpha }=\frac{a}{\sin\sigma },\;\;\frac{\rho }{\sin\beta }=\frac{a}{\sin\gamma }. \end{array}$$

If the angle equations are written in terms of the derivation of phase function, the derivative of the phase equation becomes as follows:

(20) $$\begin{array}{c} \psi \left(\phi^{\prime }\right)=a\sin\alpha -a\sin\beta . \end{array}$$

This equation is equal to 0, as long as *α* = *β* which eliminates the second term of (12).

The edge diffracted fields can be written by the aid of the quantities

(21) $$\begin{array}{c} \alpha =\alpha_{e},\;\;\beta =\beta_{e},\;\;\phi_{e}^{\prime }=\phi_{0}. \end{array}$$

In order to calculate diffraction field, the equation

(22) $$\begin{array}{c} {\vec{E}}_{d}\approx \mp {\vec{e}}_{d}\frac{1}{jk}\frac{f\left(x^{\prime }\right)}{\psi^{\prime }\left(x^{\prime }\right)}e^{-jk\psi (x\prime )} \end{array}$$

is used [4, 15]. In this equation*f*(*x*′) is expressed as magnitude, *ψ*(*x*′) is expressed as phase function and ${\vec{e}}_{d}$ is a unit vector of diffracted field. The minus and plus signs represent the upper and lower limits, respectively. In fact, this equation is the derived version of edge-point technique. The factors in this equation can be found as

(23a) $$\begin{array}{c} f_{r}\left(\phi_{0}\right)=\frac{-jkZ_{0}Ia}{4\pi }\frac{\cos\left(\left(\alpha_{e}+\beta_{e}\right)/2\right)}{\sqrt{l_{0}l_{1}}}, \end{array}$$

(23b) $$\begin{array}{c} f_{i}\left(\phi_{0}\right)=\frac{-jkZ_{0}Ia}{4\pi }\frac{\sin\left(\left(\alpha_{e}-\beta_{e}\right)/2\right)}{\sqrt{l_{0}l_{1}}} \end{array}$$

for reflected diffracted term and for incident diffracted term, respectively. The phase function and the derivative of the phase function can be also shown as

(24a) $$\begin{array}{c} \psi \left(\phi_{0}\right)=l_{0}+l_{1}, \end{array}$$

(24b) $$\begin{array}{c} \psi^{\prime }\left(\phi_{0}\right)=a\left(\sin\alpha_{e}-\sin\beta_{e}\right), \end{array}$$

where

(25a) $$\begin{array}{c} l_{0}=\rho_{0}\cos\sigma +{\text{acos}\alpha }_{e}, \end{array}$$

(25b) $$\begin{array}{c} l_{1}=\rho \cos\gamma +a\cos\alpha_{e}. \end{array}$$

The total edge diffraction is calculated as

(26) E→d=E→id+E→rd⁡=e→zZ0I8π(1sin⁡((αe−βe)/2)−1cos⁡⁡((αe+βe)/2)) ×e−jk(l0+l1)l0l1.

According to the calculation, the edge diffraction field result contains sinusoidal terms at the denominator [10]. It can be easily seen that diffracted field value diverges to infinity as

(27) $$\begin{array}{c} \sin\left(\frac{\alpha_{e}-\beta_{e}}{2}\right)\to 0,\\ \cos\left(\frac{\alpha_{e}+\beta_{e}}{2}\right)\to 0. \end{array}$$

The discontinuity can be detected on the values of transition regions. *α*_*e*_ = *β*_*e*_ is the reflection boundary, whereas *α*_*e*_ = *π* − *β*_*e*_ is the shadow boundary. Thus, the “detour parameter” method is to be used to eliminate the value equalizing the denominator to zero [16–18]. The formula of detour parameter can be shown as

(28) $$\begin{array}{c} \xi_{i,r}=-\sqrt{\psi_{i,r}-\psi_{d}}. \end{array}$$

The “detour parameter” is defined for the Fresnel function equation, and the Fresnel function can be written in terms of “detour parameter” as [18]

(29a) $$\begin{array}{c} \hat{F}\left({\hat{\xi }}_{i}\right)=F\left(\left|\xi_{i}\right|\right)\mathrm{sgn}\left(\xi_{i}\right)=\frac{e^{-j\xi_{i}^{2}-j\pi /4}}{2\sqrt{\pi }\xi_{i}}, \end{array}$$

(29b) $$\begin{array}{c} \hat{F}\left({\hat{\xi }}_{r}\right)=F\left(\left|\xi_{r}\right|\right)\mathrm{sgn}\left(\xi_{r}\right)=\frac{e^{-j\xi_{r}^{2}-j\pi /4}}{2\sqrt{\pi }\xi_{r}}, \end{array}$$

where

(30) $$\begin{array}{c} \hat{F}\left(\xi_{i,r}\right)=\frac{e^{j\pi /4}}{\sqrt{\pi }}\int_{\xi_{,i,r}}^{\infty }e^{-jt^{2}}dt. \end{array}$$

If (26) is rewritten as

(31) E→d=E→id+E→rd⁡=e→zkZ0I8π(1sin((αe−βe)/2)−1cos⁡((αe+βe)/2)) ×e−jkl0e−jkl1kl0kl1,

the conversion to the Fresnel function (*ξ*_*i*,*r*_) can be satisfied by expressing in terms of (29a) and (29b) as follows:

(32) E→d=e→zkZ0I42π(e−jkl0(2sin2((αe−βe)/2)+cos⁡⁡(αe−βe))2kl0sin⁡((αe−βe)/2)−e−jkl0(2cos⁡2((αe+βe)/2)−cos⁡(αe+βe))2kl0cos⁡((αe+βe)/2))e−jkl1kl1.

Hence the arguments of the Fresnel function can be obtained as

(33) $$\begin{array}{c} \xi_{i}=\sqrt{2kl_{1}}\sin\left(\frac{\alpha_{e}-\beta_{e}}{2}\right),\\ \xi_{r}=\sqrt{2kl_{0}}\cos\left(\frac{\alpha_{e}+\beta_{e}}{2}\right). \end{array}$$

As a result of these operations, explained previously,

(34) E→d=E→id+E→rd⁡ =e→zkZ0Iej(π/4)22πe−jkl0kl0  ×[e−jkl1cos⁡(αe−βe)F(ξi)−ejkl1cos⁡(αe+βe)F(ξr)]

uniform equations are obtained. 

## 4. Numerical Analysis

**Figure 2:**
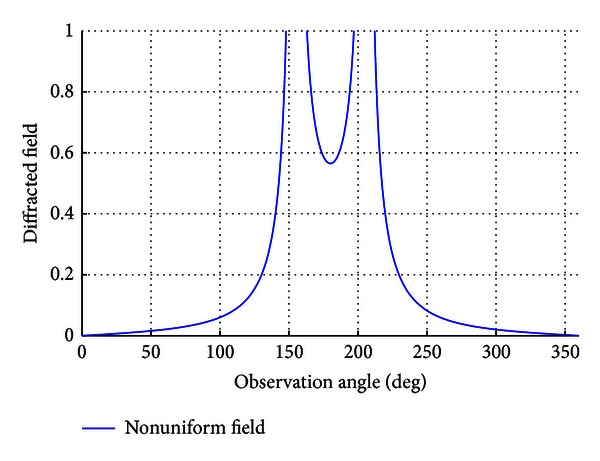
The nonuniform solution at *ϕ*_0_ = *π*/7.

The obtained results are to be compared numerically with the results of the nonuniform solution found by MTPO method. All the analyses are to be done with the value *kρ*_1_ = 100. Firstly the nonuniform field is analyzed for *ϕ*_0_ = *π*/7. As it can be seen in Figure 2, the magnitude of the diffracted fields diverges to infinity at the value *ϕ* = 15*π*/7, since sin((*α*_*e*_ − *β*_*e*_)/2) converges to 0 at that value. Also for *ϕ* = 13*π*/7, the denominator of the second term of the nonuniform solution converges to 0, as cos⁡((*α*_*e*_ + *β*_*e*_)/2) converges to 0, and the magnitude diverges to infinity. 

**Figure 3:**
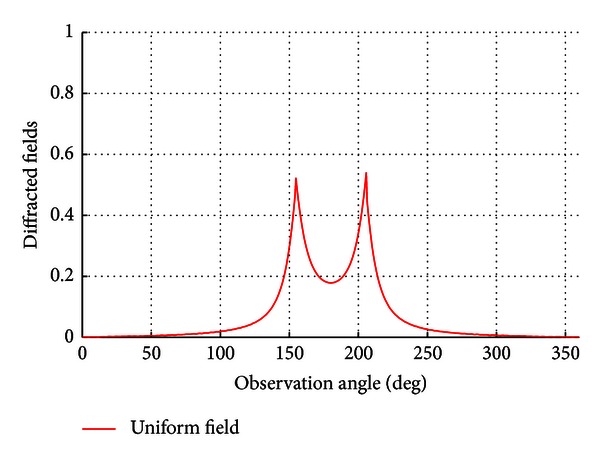
The uniform solution at *ϕ*_0_ = *π*/7.

When the “detour parameter” is applied, the uniformization of the solution leads the results to have a finite output for all values and that can be visualized in the Figure 3.

**Figure 4:**
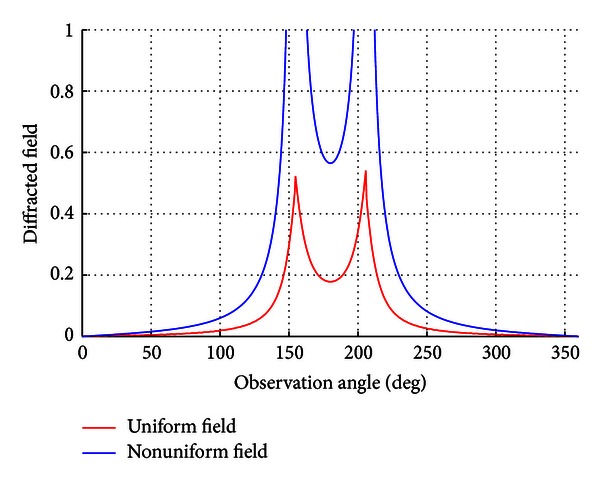
The nonuniform and uniform solution at *ϕ*_0_ = *π*/7.

As uniform and nonuniform solutions are checked together, the result in Figure 4 can be obtained.

**Figure 5:**
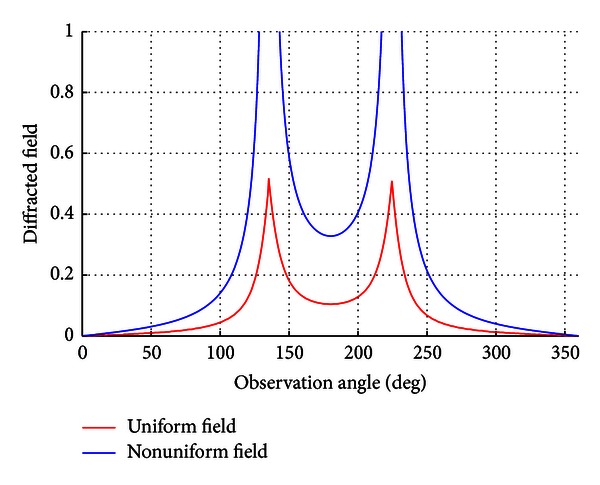
The nonuniform and uniform solution at *ϕ*_0_ = *π*/4.

Also these two types of solutions can be also analyzed for *ϕ*_0_ = *π*/4. In this analysis, for nonuniform solution at the values *ϕ* = 7*π*/4 and *ϕ* = 9*π*/4 the terms cos⁡((*α*_*e*_ + *β*_*e*_)/2) and sin((*α*_*e*_ − *β*_*e*_)/2) converge to 0, respectively, as indicated in Figure 5. The solution is continuous for the uniform solution.

## 5. Conclusion

In this study, the uniform diffracted fields are calculated on PEC cylindrical reflector with Modified Theory of Physical Optics (MTPO). It is aimed to construct a continuous solution by finding a uniform equation. MTPO theory has been explained with three axioms to construct the integral equations. The application of this theory on cylindrical electrically conducting surface has been carried out. For the diffraction the “edge-point” technique has been used, and uniform solution has been found out via “detour parameter(s).” Furthermore, the obtained results have been compared with the nonuniform ones numerically, and uniformization of the solution has been detected. Finally, by this innovative method, which has been derived in 2004, some other studies on parabolic electrically conducting surface can also be achieved in the near future.
